# Mitochondrial Targeting Involving Cholesterol-Rich Lipid Rafts in the Mechanism of Action of the Antitumor Ether Lipid and Alkylphospholipid Analog Edelfosine

**DOI:** 10.3390/pharmaceutics13050763

**Published:** 2021-05-20

**Authors:** Faustino Mollinedo, Consuelo Gajate

**Affiliations:** Centro de Investigaciones Biológicas Margarita Salas, Consejo Superior de Investigaciones Científicas (CSIC), Laboratory of Cell Death and Cancer Therapy, Department of Molecular Biomedicine, C/Ramiro de Maeztu 9, E-28040 Madrid, Spain; cgajate@cib.csic.es

**Keywords:** mitochondria, cholesterol, lipid raft, mitochondrial permeability transition pore, alkylphospholipid analog, edelfosine

## Abstract

The ether lipid edelfosine induces apoptosis selectively in tumor cells and is the prototypic molecule of a family of synthetic antitumor compounds collectively known as alkylphospholipid analogs. Cumulative evidence shows that edelfosine interacts with cholesterol-rich lipid rafts, endoplasmic reticulum (ER) and mitochondria. Edelfosine induces apoptosis in a number of hematological cancer cells by recruiting death receptors and downstream apoptotic signaling into lipid rafts, whereas it promotes apoptosis in solid tumor cells through an ER stress response. Edelfosine-induced apoptosis, mediated by lipid rafts and/or ER, requires the involvement of a mitochondrial-dependent step to eventually elicit cell death, leading to the loss of mitochondrial membrane potential, cytochrome *c* release and the triggering of cell death. The overexpression of Bcl-2 or Bcl-xL blocks edelfosine-induced apoptosis. Edelfosine induces the redistribution of lipid rafts from the plasma membrane to the mitochondria. The pro-apoptotic action of edelfosine on cancer cells is associated with the recruitment of F_1_F_O_–ATP synthase into cholesterol-rich lipid rafts. Specific inhibition of the F_O_ sector of the F_1_F_O_–ATP synthase, which contains the membrane-embedded c-subunit ring that constitutes the mitochondrial permeability transcription pore, hinders edelfosine-induced cell death. Taking together, the evidence shown here suggests that the ether lipid edelfosine could modulate cell death in cancer cells by direct interaction with mitochondria, and the reorganization of raft-located mitochondrial proteins that critically modulate cell death or survival. Here, we summarize and discuss the involvement of mitochondria in the antitumor action of the ether lipid edelfosine, pointing out the mitochondrial targeting of this drug as a major therapeutic approach, which can be extrapolated to other alkylphospholipid analogs. We also discuss the involvement of cholesterol transport and cholesterol-rich lipid rafts in the interactions between the organelles as well as in the role of mitochondria in the regulation of apoptosis in cancer cells and cancer therapy.

## 1. Introduction

**Figure 1:**
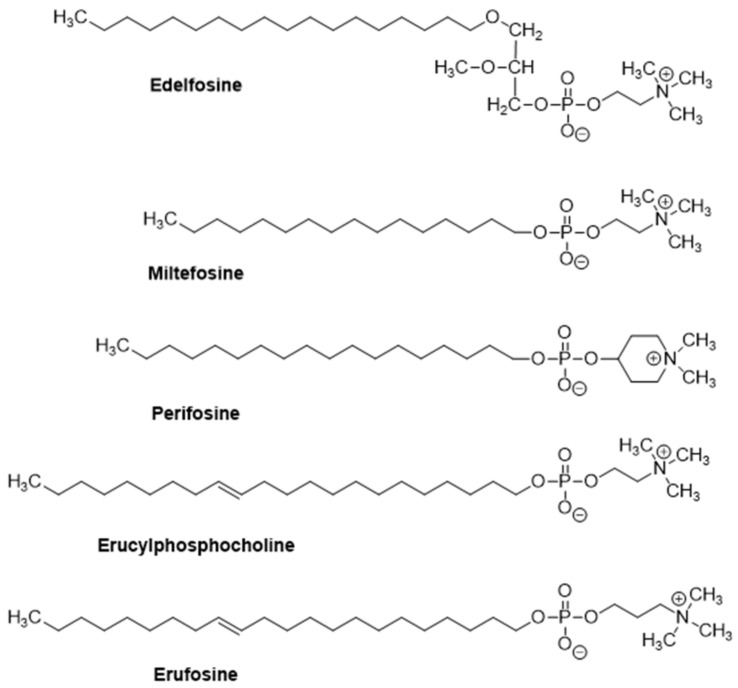
Chemical structures of some clinically relevant alkylphospholipid analogs.

The ether lipid edelfosine (1-*O*-octadecyl-2-*O*-methyl-*rac*-glycero-3-phosphocholine, ET-18-OCH_3_) (Figure 1) is considered as the prototype of a family of synthetic antitumor drugs collectively known as alkylphospholipid analogs (APLs) or antitumor ether lipids (AELs) [1,2,3]. Among the distinct APLs, it is worth highlighting miltefosine, perifosine, erucylphosphocholine and erufosine, in addition to edelfosine (Figure 1). Miltefosine (hexadecyl 2-(trimethylazaniumyl)ethyl phosphate, also known as hexadecylphosphocholine) represents the minimal structural requirement for the antitumor activity of APLs and has become the first oral drug in the treatment of visceral leishmaniasis [4,5,6], being commercialized under the trademark name of Impavido^®^(oral solid human pharmaceutical product; Zentaris, Frankfurt, Germany). Miltefosine is also used in the clinic as a topical treatment for cutaneous metastases of breast cancer [7], and commercialized under the trademark name of Miltex^®^ (topical liquid human pharmaceutical product; Baxter, Newbury, UK). Miltefosine is also used under the trademark of Milteforan^®^ for the treatment of canine leishmaniasis (oral liquid veterinary pharmaceutical product for dogs; Virbac, Carros, France) [8]. Perifosine (octadecyl-[1,1-dimethyl-piperidino-4-yl]phosphate), where the choline moiety of miltefosine is replaced by a heterocyclic piperidine group, shows a promising orally active antitumor APL [9,10] that is currently used in clinical trials [11,12,13,14,15]. Erucylphosphocholine ([13Z]-docos-13-en-1-yl 2-(trimethylammonio)ethyl phosphate, ErPC), an APL-derivative with a 22 carbon atom chain and a *cis*-13, 14 double bond, shows distinctive reduced hemolytic activity, thereby allowing intravenous injection, and holds promise for the treatment of human brain tumors [16,17,18,19]. The ErPC closely related congener erufosine (erucylphosphohomocholine, or erucylphospho-*N*,*N*,*N*-trimethylpropylammonium, ErPC3) [20,21], a member of the latest generation of APLs, can be applied intravenously and can cross the blood–brain barrier [22,23,24,25].

However, edelfosine remains as the most active antitumor APL, and is the golden standard and prototype for other APLs and for studies on the mechanism of action of this family of compounds. Furthermore, our in vitro and in vivo results have revealed that edelfosine, orally administered, is the most potent APL in killing different *Leishmania spp.*, showing higher anti-*Leishmania* activity than miltefosine, and is less prone to generate drug resistance than miltefosine [26].

A major feature of the above APLs is that they target cell membranes, particularly lipid rafts, affecting several biochemical processes, ion transport and signaling pathways [1,2,27,28,29,30]. Edelfosine shows a high affinity for both model and cell membranes, but weak detergent activity [31]. A remarkable characteristic of the ether lipid edelfosine is its selectivity in inducing apoptosis in cancer cells, whereas non-transformed cells are spared [1,2,9,27,32]. This selectivity is due to the preferential drug uptake by cancer cells by a not yet fully understood mechanism [1,2,27,33,34,35]. Edelfosine targets the cell membrane and, depending on the cell type, leads to the onset of different types of cell death [36], ranging from apoptosis, which is predominantly triggered in most cancer cells [1,2,27,33,34,35], to necrosis/necroptosis [37,38], with mitochondria playing a key role in the irreversible onset of the cell death process [9,39,40].

## 2. The Alkylphospholipid Analog Edelfosine Induces Apoptosis Selectively in Cancer Cells

A direct antitumor action of edelfosine on cancer cells was already reported in the late 1970s and 1980s [41,42,43,44], but it was not until the 1990s and 2000s that the molecular mechanism underlying the antitumor activity of this drug started to be unveiled, showing that the induction of apoptosis by edelfosine was the main effect that explained the direct antitumor action of APLs [45,46]. Then, a number of findings in the Faustino Mollinedo’s and Consuelo Gajate’s laboratory, first in Valladolid (Spain) and then in Salamanca (Spain) in the late 1990s and early 2000s, respectively, demonstrated the selective pro-apoptotic effect of edelfosine on cancer cells, following the preferential drug uptake in tumor cells [32,33,47] and the reorganization of membrane lipid raft platforms [27,48]. These data provided the first evidence for a selective pro-apoptotic drug and for the involvement of lipid rafts in cancer chemotherapy. Edelfosine (Figure 1) is an oral drug showing potent antitumor activity against different kinds of tumors in cancer animal models [34,35,40,49], and lacks toxicity in rats after edelfosine oral treatment at pharmacological relevant doses, with no cardiotoxicity, hepatotoxicity or renal toxicity [50].

In general, apoptosis can be mainly induced either by an extrinsic pathway, mediated through the activation of death receptors, or by an intrinsic pathway or mitochondria-mediated process, which permeabilizes the outer mitochondrial membrane (OMM), leading to the release of cytochrome *c*, located in the mitochondrial intermembrane/intercristae spaces where it functions as an electron shuttle in the respiratory chain. Mitochondria-mediated apoptosis is characterized by mitochondrial outer membrane permeabilization (MOMP) and the subsequent release of mitochondrial cytochrome *c* into the cytoplasm to activate caspases. Once in the cytosol, cytochrome *c* binds the adaptor molecule APAF-1 (apoptosis protease-activating factor-1), causing it to oligomerize through a conformational change, and form a heptameric structure called the apoptosome complex, made up of cytochrome *c* and APAF-1. The apoptosome recruits and potentiates the activation of procaspase-9, which in turn cleaves and activates downstream effector caspases, such as caspase-3 and -7 [51]. MOMP is regulated by the Bcl-2 family of proteins [51].

## 3. Edelfosine Accumulates in Lipid Rafts and the Endoplasmic Reticulum of Cancer Cells, and the Generated Apoptotic Signals Converge on Mitochondria to Elicit Apoptosis

A major milestone in the study of the mechanism of action of APLs was achieved in 2001 when the apoptosis induced by the ether lipid edelfosine was first found to be mediated by lipid rafts [48]. Edelfosine accumulates in the lipid rafts of a wide array of hematological cancer cells [9,27,34,52,53,54], leading to apoptosis through the reorganization of these membrane domains, especially by promoting co-clustering of lipid rafts and Fas/CD95 death receptor signaling [9,27,34,53,54]. These seminal reports identified lipid rafts as a novel and promising target in cancer therapy [9,27,34,53,54,55,56,57], and paved the way for future studies in raft-targeted cancer therapy [29,30,56,58,59,60,61,62,63]. Edelfosine induced the clustering and recruitment of the Fas/CD95 death receptor, as well as other death receptors and downstream signaling molecules in lipid rafts, thus triggering apoptosis in a variety of cancer cells, including myeloid and lymphoid cancer cells [9,27,33,48]. This mechanism of action involved a raft-mediated activation of apoptosis via Fas/CD95, independently of its physiological FasL/CD95L ligand, which could be pharmacologically modulated, thus opening a new therapeutic approach in cancer therapy [1,27,34,35,53,59,64]. Interestingly, edelfosine prompted the recruitment of death receptors and downstream signaling molecules in lipid rafts, whereas Akt survival signaling was displaced from the rafts [30,65].

Yeast cells show different raft domains that contain transporters and proteins involved in the control of Na^+^, K^+^ and pH homeostasis, required for the proper function of yeast, and that modulate yeast growth and death [66]. The active transport of ions and nutrients in yeast relies on the existence of an electrochemical gradient of protons across the plasma membrane, and this electrochemical gradient is mainly generated in *Saccharomyces cerevisiae* by the H^+^-ATPase gene *pma1*, an essential H^+^ pump for yeast growth [67] and a resident raft protein [68]. We found that edelfosine treatment in *S. cerevisiae* displaced Pma1p from lipid rafts [69,70,71], and induced its internalization as well as of the plasma membrane arginine/H^+^ symporter Can1p (arginine permease) and the uracil/H^+^ symporter Fura4p (uracil permease), two nutrient H^+^-symporters associated with yeast lipid rafts [66,72,73]. Our studies on the mechanism of action of edelfosine in *S. cerevisase* showed that the ether lipid displaces the essential proton pump Pma1p from the lipid rafts, inducing its internalization into the vacuole, the yeast equivalent to the mammalian endosome–lysosome system, and subsequent degradation, thus leading to altered pH homeostasis and cell death [69,70,71]. The displacement of Pma1p from the rafts following edelfosine treatment was preceded by the rapid movement of the yeast sterol ergosterol out of the plasma membrane and into the cell [69,70,71].

Taking together, edelfosine induces cell death through the reorganization of lipid rafts by modifying the balance of apoptotic versus survival signaling molecules in these membrane domains. Thus, the recruitment of apoptotic signaling molecules into lipid rafts and the displacement of survival molecules from these membrane domains is critical in the mechanism of action of this ether lipid.

Biophysical studies have shown that edelfosine has a high affinity for cholesterol, increases membrane thickness, and alters raft organization [74]. The high affinity of edelfosine for cholesterol is easily and visually explained on the basis of the complementarity of the molecular geometries of edelfosine and sterols in general [75]. The combination of “cone-shape” sterols and “inverted cone-shape” edelfosine leads to a more stable bilayer [75].

Studies on solid tumor cells, including pancreatic adenocarcinoma, lung adenocarcinoma, cervix epithelioid carcinoma and Ewing’s sarcoma cells, have shown that edelfosine accumulated mainly in the endoplasmic reticulum (ER), triggering an ER stress response that eventually led to apoptosis [39,40,49,76]. Interestingly, edelfosine accumulated first in plasma membrane lipid rafts and subsequently in the ER of *S. cerevisiae*, used as a eukaryotic model organism [70].

**Figure 2:**
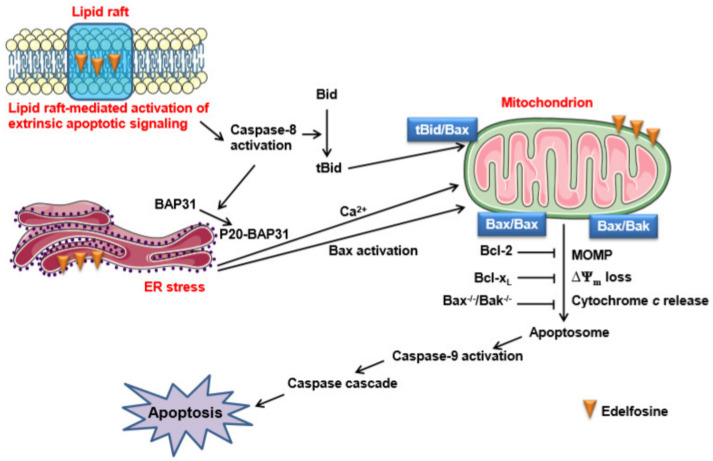
Schematic model of the involvement of plasma membrane lipid rafts, ER and mitochondria in edelfosine-induced apoptosis in cancer cells. Protection of mitochondria by Bcl-2 or Bcl-xL overexpression, or by Bax/Bak double knock-out (Bax^-/-^/Bak^-/-^), prevents cell death, indicating that the apoptotic signals derived from plasma membrane and ER converge on mitochondria. See text for details. MOMP, mitochondrial outer membrane permeabilization.

However, although edelfosine has been found to accumulate in the membrane rafts [9,27,34,53,54] and the ER [40,49] of human tumor cells, as assessed by using radioactive edelfosine and fluorescent analogs of the ether lipid, all the apoptotic signals generated from either the plasma membrane rafts or the ER converge on the mitochondria to eventually trigger apoptosis (Figure 2). Thus, the overexpression of Bcl-2 or Bcl-x_L_ totally prevents the apoptotic response induced by edelfosine in cancer cells, either from a hematological or solid tumor origin [9,32,40,77]. These data highlight the critical role of mitochondria as a meeting and convergence point of different apoptotic signaling pathways, irreversibly leading to apoptosis.

Edelfosine-induced apoptosis involved mitochondria as assessed by the disruption of the mitochondrial transmembrane potential (ΔΨ_m_), measured using 3,3′-dihexyloxacarbocyanine iodide [DiOC6(3)] fluorescence, and the production of reactive oxygen species (ROS), detected using the conversion of dihydroethidium into ethidium, in both leukemic [47] and solid tumor cells [49]. Edelfosine also induced Bax activation, cytochrome *c* release, caspase-9 activation, and DNA fragmentation in both leukemic [9,47] and solid tumor cells [40,49], and Bcl-2 or Bcl-x_L_ overexpression prevented the above-mentioned mitochondria-related responses [9,40,77].

## 4. Localization of Edelfosine in the Mitochondria of Cancer Cells Using Fluorescent Analogs

**Figure 3:**
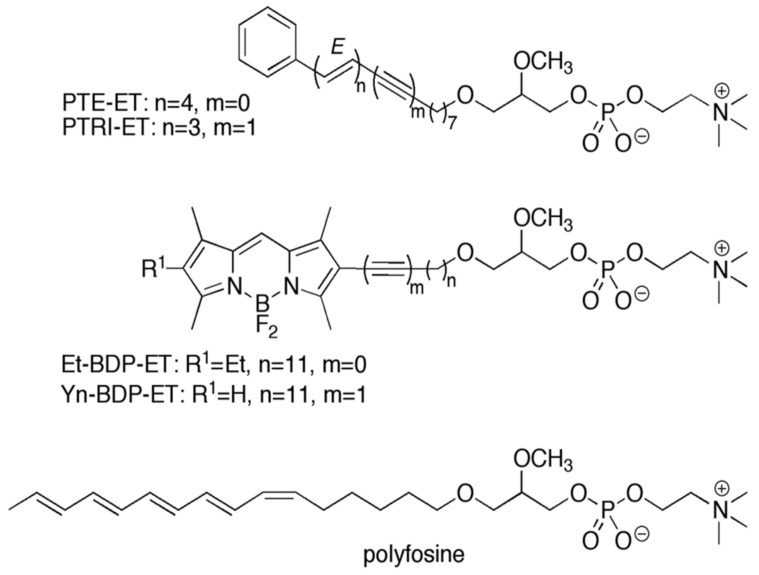
Chemical structures of fluorescent edelfosine analogs.

**Figure 4:**
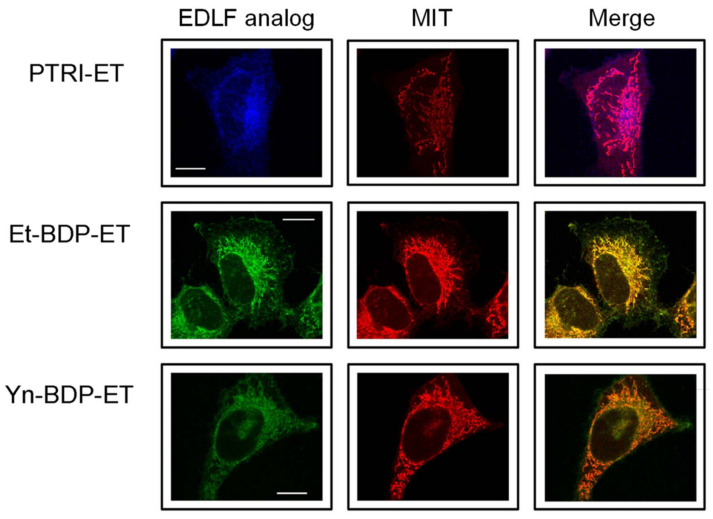
Colocalization of fluorescent edelfosine analogs and mitochondria in cancer cells. HeLa cells were incubated for 12 h with 10 μM of the indicated fluorescent edelfosine (EDLF) analogs (PTRI-ET, blue fluorescence; Et-BDP-ET, green fluorescence; Yn-BDP-ET, green fluorescence) to visualize edelfosine subcellular localization. Mitochondrial location was examined using MitoTracker Red probe (MIT, red fluorescence). Areas of colocalization between edelfosine analogs and mitochondria in the merge panels are purple (for PTRI-ET) or yellow (for Et-BDP-ET and Yn-BDP-ET). Bar, 10 μm. Image taken from [81], Springer Nature, 2011.

In 2004, we synthesized the first fluorescent edelfosine analog, which preserved pro-apoptotic activity comparable to that of the parent drug [27,78], as an excellent tool to unveil the mechanism of action of this drug. To this aim, we tried to synthesize a fluorescent edelfosine analog with a minimum modification of the chemical structure. Our previous structure–activity relationship studies at the time showed that some modifications preserved the apoptotic activity, including the presence of a double bond in the *O*-octadecyl chain at the C1 of edelfosine [32]. In this regard, a conjugated pentaene group appeared as a convenient candidate, considering that this fluorophore had led to the development of useful fluorescent probes for lipid membranes [79,80]. On these grounds, we reasoned that the replacement of the C18 aliphatic chain by a lipophilic fluorescent group of similar length could preserve the unique properties of this drug regarding its activity and selectivity. This led to the synthesis of the first fluorescent analog, containing the conjugated all-(*E*)-phenyltetraene blue-emitting chromophore, which was coined as PTE-ET [27] (Figure 3). This PTE-ET fluorescent analog, as well as the subsequently synthesized PTRI-ET (Figure 3), containing the all-(*E*)-phenyltrienyne blue-emitting chromophore, were the first fluorescent edelfosine analogs [27,70,78,81]. These fluorescent edelfosine analogs largely preserved the chemical structure of edelfosine (Figure 3), and shared analogous fluorescence traits with a poor fluorescence yield and photostability under intense near-UV laser excitation [28,78,81]. In order to improve the fluorescence yield and provide a more stable fluorescent signal, we synthesized a second generation of fluorescent analogs by adding a BODIPY (4,4-difluoro-4-bora-3a,4a-diaza-s-indacene; boron-dipyrromethene) fluorochrome attached to the alkyl chain of edelfosine, leading to the green-emitting Et-BDP-ET and Yn-BDP-ET fluorescent edelfosine analogs [28,81] (Figure 3). These two compounds had a higher fluorescence yield and resistance to photodegradation than the first generation fluorescent edelfosine analogs, and allowed a thorough analysis through confocal microscopy [28,81]. The use of all the above fluorescent edelfosine analogs, either first or second generation, allowed to localize edelfosine in the mitochondria of cancer cells (Figure 4) [81,82], in addition to the subcellular localizations of this drug in the ER [40,49,76] and lipid rafts [9,27,34,35] in solid tumor cells and hematological cancer cells, respectively. Interestingly, mitochondrial localization of edelfosine was also found in *Leishmania* parasites [82].

Polyene lipids (linear hydrocarbons containing a conjugated double-bond system) display a unique structural similarity to natural lipids, which results in minimal effects on the lipid properties. The above PTE-ET fluorescent analog could be included in this type of lipids. In this regard, polyfosine (Figure 3), a polyene fluorescent analog of edelfosine containing five conjugated double bonds, was also found to accumulate in the mitochondria and to induce morphological changes and apoptosis in COS7 cells [83].

## 5. Cholesterol in Mitochondria

A major question raises from the above subcellular localization experiments. How does edelfosine accumulate in the mitochondrial membrane? We reason that a putative explanation for this accumulation could lie in the above-stated high affinity of edelfosine for cholesterol.

Lipids are not randomly distributed among biological membranes, but their relative content is characteristic for each organelle, affecting their shape, structure and function [84]. Lipids constitute approximately 50% of the mass of most cell membranes (e.g., plasma membrane), although this proportion is highly dependent on the type of membrane (e.g., mitochondrial inner membrane contains 75% protein as a result of the abundance of protein complexes involved in electron transport and oxidative phosphorylation). However, one must bear always in mind that there are many more lipid molecules than protein molecules in membranes because lipid molecules are small compared with proteins. On these grounds, it might be estimated the presence of about 50 lipid molecules for each protein molecule in the plasma membrane.

Among the distinct lipids, cholesterol (a major sterol component in animal cell membranes, making up about 30% of the lipid bilayer on average) has attracted much attention since its first isolation from gallstones in the eighteen century. The French doctor and chemist François-Paul Poulletier de la Salle (1719–1788) first identified cholesterol in gallstones in about 1758–1769, albeit his work was never published [85,86,87]. Later on, cholesterol was rediscovered in 1815 by the French chemist Michel Eugène Chevreul (1786–1889) who named it “cholesterine” [85,86,87].

Cholesterol is an essential building block of the plasma membrane, having diverse structural and functional roles [88,89], and playing pleiotropic actions in normal and cancer cells [30]. Cholesterol plays a unique and pivotal role among the different lipids in maintaining the structural integrity and regulating the fluidity of the mammalian cell membranes [90,91]. As compared to other lipids, cholesterol moves rapidly as a monomer across membranes and between membrane organelles on protein carriers [89]. However, cholesterol is not uniformly distributed within biological membranes and across different cellular compartments. Cholesterol has been suggested to be enriched in the cytosolic (inner) leaflet of the plasma membrane [92]. Recent imaging methods, using tunable orthogonal cholesterol sensors, have revealed a marked transbilayer asymmetry of plasma membrane cholesterol in mammalian cells, with the cholesterol concentration in the inner leaflet being ~12-fold lower than in the outer leaflet [93]. Cellular cholesterol, derived from low-density lipoprotein receptor-mediated endocytosis or synthesized de novo in the ER, is mainly (up to 90%) located in the plasma membrane, constituting 10–45% (mol%) of the total plasma membrane lipids [93,94,95]. Cholesterol plays major roles in the structural and functional modulation of integral membrane proteins [96], and in the formation of cholesterol-rich membrane domains, such as the so-called lipid or membrane rafts. Lipid rafts are membrane microdomains enriched in cholesterol and sphingolipids, involved in the lateral compartmentalization of molecules at the cell surface, and can coalesce to form membrane raft platforms [30,97].

Mitochondrial membranes are cholesterol-poor, particularly the inner mitochondrial membrane, as compared to other subcellular membranes in mammalian cells [98,99]. The relative proportion of phospholipid/cholesterol in the rat liver plasma membrane is 5.25, whereas this rate increases up to 58.3 in the rat liver mitochondria [98,99]. The sterol-to-protein ratio in mitochondria is low compared to other subcellular fractions (rat liver), as follows: mitochondria (0.003 mg sterol/mg protein); ER (0.014 mg sterol/mg protein); lysosomes (0.038 mg sterol/mg protein); Golgi (0.038 mg sterol/mg protein); and plasma membrane (0.128 mg sterol/mg protein) [98,99].

**Figure 5:**
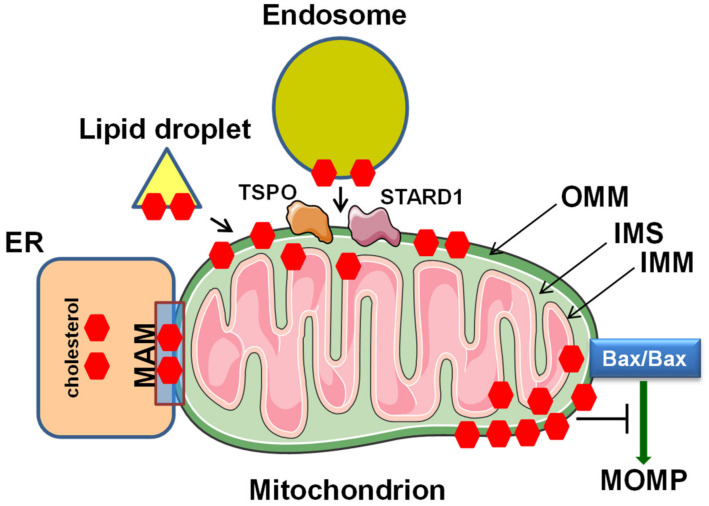
Import and transfer of cellular cholesterol into mitochondria. Cholesterol is transported to the mitochondria through vesicular and non-vesicular trafficking, involving the ER, lipid droplets, endosomes, TSPO and STARD1. Elevated mitochondrial cholesterol levels in cancer cells affect mitochondrial membrane and impair Bax/Bak oligomerization in OMM and subsequent MOMP formation, representing a mechanism of cell death resistance in tumor cells. See text for details. ER, endoplasmic reticulum. IMM, inner mitochondrial membrane. IMS, inter-membrane space. MAM, mitochondria-associated membrane. OMM, outer mitochondrial membrane. STARD1, steroidogenic acute regulatory protein-related lipid transfer domain containing protein 1. TSPO, translocator protein.

The mitochondria are made up of an OMM, an inner mitochondrial membrane (IMM), an inter-membrane space (IMS) in between, and the mitochondrial matrix enclosed by the IMM (Figure 5). The IMM shows a number of invaginations, called cristae, thus making the surface of the IMM significantly larger than that of the OMM. The whole machinery of oxidative phosphorylation, including the electron transport chain (ETC) complexes (complexes I–IV) as well as the F_1_F_O_–ATP synthase (complex V), resides in the IMM. The OMM separates the mitochondrion from the cytosol. The OMM forms a smooth lipid-rich surface with high membrane fluidity, whereas the IMM is highly folded and shows an elevated protein level and lower lipid content [98,99]. In this regard, cholesterol is enriched in the OMM compared to the IMM (0.04 mg sterol/mg protein in OMM versus <0.01 mg sterol/mg protein in IMM, rat liver) [98,99].

As stated above, cholesterol levels vary widely between different subcellular membranes (e.g., plasma membrane contains about 40-fold higher cholesterol levels than the ER and mitochondria [98,99]). Although cholesterol levels are particularly low in mitochondria, especially in the IMM [98,99], cholesterol must reach this subcellular compartment for the correct functioning of several major biological processes, including the synthesis of steroids, oxysterols and bile acids.

This variety in cholesterol content between different biological membranes is consistent with its putative major role in the regulation of the correct functioning of the distinct subcellular organelles. The low level of cholesterol in the mitochondria suggests that even small changes in its concentration, either through a general increase in sterol content or a particular clustering of cholesterol in certain membrane regions, could have a large impact on the biophysical and functional features of the membrane and organelle.

Cholesterol critically influences membrane fluidity, permeability, curvature and membrane protein interactions in biological cell membranes [30,60,100,101,102], affecting the cell surface distribution of membrane proteins, modulation of cellular signaling transmission and intracellular trafficking. A major feature of cholesterol is its ability to modulate the physicochemical properties of cellular membranes. Cholesterol orients in a phospholipid bilayer with its polar hydroxyl group towards the aqueous phase and the hydrophobic steroid ring oriented parallel to the hydrocarbon chains of the phospholipids, thus interacting with the membrane phospholipids and sphingolipids and being a critical contributor to lipid raft assembly [103].

## 6. Cholesterol Transport to Mitochondria

The insertion of cholesterol into the membrane lends rigidity and promotes the formation of protein-tethering platforms, such as lipid rafts [30,97]. In the mitochondria, cholesterol plays a number of major roles, some are as follows [104]: (a) a structural component of the OMM and IMM, providing the appropriate fluidity, curvature and biophysical properties; (b) a precursor of steroidogenesis, by which cholesterol is converted to biologically active steroid hormones, with the first biochemical reactions taking place in the mitochondrial matrix [105]; (c) the core of membrane platforms interacting with the ER, lysosomes and other vesicles or intracellular compartments; and (d) a tethering element for mitochondrial DNA. 

Cholesterol is transported to the mitochondria through vesicular and non-vesicular trafficking. Some critical proteins and organelles/vesicles involved in mitochondrial cholesterol delivery to the mitochondria are schematically displayed in Figure 5, and indicated below.

STARD1 (30 kDa steroidogenic acute regulatory protein-related lipid transfer domain containing protein 1) acts at the OMM to mediate the import of cholesterol and transports cholesterol from the OMM to the IMM [106].

Translocator protein (18 kDa), TSPO (formerly known as the peripheral-type benzodiazepine receptor), is a ubiquitous mitochondrial protein, localized to the OMM, and involved in several biological functions, including mitochondrial cholesterol transport and steroid hormone biosynthesis [107,108]. TSPO is a five transmembrane domain protein found as a monomer, dimer and polymer, and is highly abundant in the OMM [107,108]. TSPO has been shown to interact with STARD1 and the voltage-dependent anion-selective channel 1 (VDAC1), the latter being the most abundant VDAC of the three isoforms VDAC1-3 [109,110]. TSPO is a high-affinity cholesterol-binding protein that oligomerizes to form a cholesterol transporting channel and prompts cholesterol transfer to the IMM [111,112]. TSPO has been shown to associate with different cytosolic and mitochondrial proteins as part of a large multiprotein complex involved in mitochondrial cholesterol transport [108,109].

Cholesterol transfer to the mitochondria is mediated by a series of direct interactions between the mitochondria and a series of intracellular organelles, such as ER, lipid droplets and endosomes (Figure 5). The mitochondria and the ER interact through the so-called mitochondria-associated membranes (MAMs) [113], which are involved in the transfer of cholesterol and other lipids between the ER and mitochondria [114]. Wide-field fluorescence microscopy combined with digital deconvolution has revealed that mitochondria form a largely interconnected dynamic network, and by expressing different fluorescent markers targeted to the mitochondria and ER, 5–20% of the mitochondrial surface was estimated to be in close apposition to (10–30 nm distance) or in association with the ER [115]. In fact, a large body of evidence demonstrates that mitochondria interact and communicate directly with the ER through MAMs to modulate several cellular responses [115,116,117,118,119,120,121].

Lipid droplets, originating from the ER, are dynamic structures able to interact with most other cellular organelles, are critical to buffer the levels of toxic lipid species [122,123] and are involved in lipid storage and mobilization [123]. Lipid droplets have been envisaged to interact directly with mitochondria to facilitate lipid transfer [124,125]. These lipid droplet–mitochondria interactions have been suggested to be mediated by SNAP-23 (23-kDa synaptosome-associated protein) [126], a protein that plays a major role in general membrane fusion processes, and serves as an important regulator of transport vesicle docking and fusion in all mammalian cells [127,128,129,130].

A major source of cholesterol is derived from the endocytosis of exogenous lipoproteins, transferring cholesterol from the lipoproteins and plasma membrane to the endosomes and multivesicular late endosomes [131,132,133]. The subsequent transport of cholesterol out of late endosomes requires the so-called Niemann-Pick type C1 (NPC1) and NCP2 proteins. Niemann-Pick disease type C (NP-C) is a rare neurodegenerative disorder of autosomal recessive inheritance, with an estimated incidence of 1 in 120,000–150,000 live births, and affects cholesterol trafficking [134,135]. NP-C is characterized by endosomal accumulation of unesterified cholesterol and glycolipids in various tissues, including the brain, leading to progressive central nervous system neurodegeneration and death [135]. This disease is caused by mutations of the *NPC1* (accounting for 95% of NP-C cases) or the *NPC2* gene (5% of NP-C cases). Currently, there is no cure for NP-C and patients usually die before adulthood (frequently in the second decade of life), but adult forms of NP-C are being increasingly recognized, having a more insidious onset and slower progression [136,137]. NP-C is characterized by impaired cholesterol efflux from late endosomes and lysosomes, and secondary accumulation of lipids due to mutations in the NPC1 or NPC2 proteins, which act in coordination to mediate the efflux of unesterified cholesterol from lysosomes or late endosomes. Human NPC1 encodes a 1278 amino acid (170–190 kDa) glycoprotein, found in late endosomes and lysosomal membranes, with 13 transmembrane domains, which binds both cholesterol and oxysterol [138]. NPC2 (18 kDa) is a soluble lysosomal glycoprotein containing 132 amino acids and is found in the lumen of late endosomes/lysosomes [139,140]. NPC1 binds cholesterol with nanomolar affinity, whereas NPC2 binds cholesterol with micromolar affinity [138,141]. In relation to this review, it is interesting to note that resistance to the ether lipid drug edelfosine represents the first phenotype caused by the deletion of the *NCR1* gene in *S. cerevisiae* [142], further supporting the strong relationship between the ether lipid edelfosine and cholesterol. *NCR1* is the *S. cerevisiae* homolog of the human *NCP1*, and the Ncr1p protein localizes to the vacuole [142]. Under normal circumstances, NPC2, as a soluble sterol transfer protein in the late endosome, transfers cholesterol from the internal vesicle to the membrane-bound NPC1, which mediates cholesterol egress from the late endosomes to the ER and plasma membrane. Putative transport from the late endosome to the mitochondria could be mediated through STARD3 (also known as metastatic lymph node 64 protein (MLN64)), a 50.5 kDa protein (containing 445 amino acids) that localizes in the membrane of late endosomes, and is involved in cholesterol transport [143,144]. However, the direct transport from endosomes to mitochondria or through an ER-mediated step is still a matter of controversy.

## 7. Mitochondrial Cholesterol in Cancer

Mitochondria are considered cholesterol-poor organelles, with estimates ranging from 0.5–3% of the content found in the plasma membrane [145]. However, increased mitochondrial cholesterol levels have been reported in a number of diseases or pathophysiological conditions, including some types of cancer, steatohepatitis, cardiac ischemia, aging and neurodegenerative diseases [146]. When it comes to neurodegenerative diseases, Alzheimer’s disease and the lysosomal disorder NP-C call particular attention [147]. The functions of mitochondria are altered in all of the above conditions, and it is tempting to suggest the existence of an interplay between the abnormally increased mitochondrial cholesterol levels, mitochondria dysfunction and disease pathology [146,147]. The accumulation of intracellular cholesterol alters mitophagy and reduces the clearance of defective mitochondria in neurodegenerative diseases [148]. Regarding cancer, larger amounts of mitochondrial cholesterol have been found in solid tumors as compared to their normal counterparts, and this correlates with tumor growth and malignancy [149]. About 2- to 5-fold higher levels of mitochondrial cholesterol were found in the tumors from Buffalo rats containing transplanted Morris hepatomas, when compared to the content found in the mitochondria prepared from a host liver [150,151]. The mitochondrial cholesterol levels in H35 rat hepatoma cells and HepG2 human hepatoma cells were 3- to 10-fold higher than the corresponding cholesterol levels in normal rat and human liver mitochondria [152]. The high levels of mitochondrial cholesterol contribute to chemotherapy resistance [149,152].

As stated above, cholesterol level tends to be high in cancer cells, the meaning of which is currently controversial [30,153,154]. A number of studies have shown elevated mitochondrial cholesterol levels in cancer cells, being associated with chemotherapy resistance, low mitochondrial proton leak, and altered patterns of the Krebs cycle metabolism, which might affect the activity of certain mitochondrial enzymes [150,151,152,155,156]. An increased cholesterol level in the OMM, and its subsequent decrease in membrane fluidity, inhibits Bax oligomerization and activation (Figure 5), thus impairing MOMP and contributing to the resistance to apoptosis-inducing agents [149,157].

## 8. Mitochondrial Permeability Transition Pore (mPTP) and Regulation of Cell Death

Mitochondria are critical subcellular structures that control cellular life through energy production as well as cell death through the induction of apoptosis and necrosis. Different death signaling pathways converge on mitochondria, and the so-called mitochondrial permeability transition pore (mPTP) acts as a key nodal point in mediating cell death. Mitochondrial permeability transition is defined as the process whereby the IMM shows an increased permeability to solutes with a molecular mass of <1.5 kDa, thus resulting in the loss of the IMM potential, respiratory chain uncoupling, halt of mitochondrial ATP synthesis, mitochondrial swelling, OMM rupture, and eventually cell death [158,159,160,161,162]. The molecular identity of the mPTP is rather controversial, and different proteins have been suggested to be part of the mPTP complex or closely related to its function as regulators of mPTP activity. These proteins include the following: the adenine nucleotide translocator (ANT) [163], a 32 kDa protein located in the IMM responsible for the import of ADP into the mitochondrial matrix in exchange for ATP; VDAC1, the most abundant protein in the OMM with a molecular weight of ~32 kDa through which metabolites and nucleotides traverse the OMM [164]; the translocator protein (TSPO) (also known as the peripheral benzodiazepine receptor) [165], a 18 kDa transmembrane protein mainly found on the OMM [166], which is required for the mitochondrial cholesterol import that is essential for steroid hormone production [167]; the mitochondrial phosphate carrier (PiC) (also known as SLC25A3; solute carrier family 25, member 3), a ~40 kDa IMM solute carrier that is the primary transporter of inorganic phosphate (Pi) into the mitochondrial matrix [168]; and cyclophilin D (CypD), a 18.9 kDa matrix peptidyl-prolyl cis-trans isomerase that resides in the mitochondrial matrix, associates with the inner mitochondrial membrane during the mitochondrial membrane permeability transition [161], and interacts with and modulates F_1_F_O_–ATP synthase [169,170].

However, subsequent genetic studies showed the mPTP opening in the absence of ANT, VDAC, TSPO and PiC, suggesting that these proteins are not an integral component of the mPTP structure, but rather may play regulatory roles in pore formation [171,172,173,174,175].

The evidence accumulated in the last ten years has brought a new player to the scene that provides the key to the solution of the elusive and long-lasting enigma in mPTP biology. This new player is F_1_F_O_–ATP synthase, the ubiquitous and universal enzyme that provides cellular energy in the form of ATP by oxidative phosphorylation and photophosphorylation in animals, plants and microorganisms, thus leading to a dual and critical role of F_1_F_O_–ATP synthase in the regulation of cell life and death, playing major roles in energy generation and apoptosis regulation [176].

In mitochondria, oxidative phosphorylation has the following two critical parts: the ETC and chemiosmosis. The ETC includes a series of protein complexes (complex I, II, III and IV) bound to the IMM, through which electrons pass through in a series of redox reactions, leading to the translocation of protons from the mitochondrial matrix to the IMS, and thus forming an electrochemical gradient. This proton gradient increases the acidity in the IMS, generating an electrical difference with a positive charge outside and a negative charge inside. F_1_F_O_–ATP synthase (also known as complex V) uses the ETC-generated proton gradient across the IMM to form ATP through a chemiosmotic process. This enzyme is made up of two mechanical rotary motors, each driven by ATP hydrolysis or proton flux down the membrane potential of the protons. These two molecular motors, connected by a common rotor shaft, interconvert the chemical energy of ATP hydrolysis and proton electrochemical potential through mechanical rotation of the rotary shaft.

**Figure 6:**
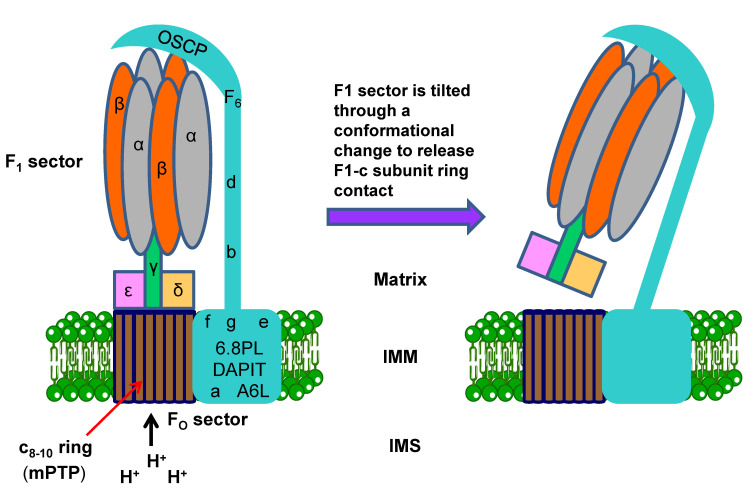
Organization of subunits in F_1_F_O_–ATP synthase in mammalian mitochondria and opening of the mPTP. OSCP, F6, d, and b constitute the F_O_ peripheral stalk. Under certain circumstances, the F_O_ peripheral stalk and F_1_ sector are tilted to free the c-subunit ring channel from the side facing the matrix, thus opening the mPTP (see [190]). See text for details. IMM, inner mitochondrial membrane. IMS, inter-membrane space. mPTP, mitochondrial permeability transition pore.

Mitochondrial F_1_F_O_–ATP synthase can undergo a Ca^2+^-dependent transformation to form channels with properties matching those of the mPTP, as a key player in cell death [177]. The catalytic site of the F_1_F_O_–ATP synthase β subunit constitutes the Ca^2+^ trigger site, involved in the induction of a conformational change and transition of the F_1_F_O_–ATP synthase to a channel, behaving as an mPTP [177]. F_1_F_O_–ATP synthase is a complex enzyme with a molecular weight of >500 kDa, made up of two sectors, the inner membrane bound F_O_ region (indicating that it can be inhibited by the antibiotic oligomycin) and the matrix-exposed F_1_ region, acting as rotary motors (Figure 6). The F1 sector (~380 kDa) is the hydrophilic water-soluble part of the complex, which acts as an ATP-driven motor and is composed of three copies of each of the subunits α and β (catalytic subunit), forming the catalytic head of the complex, and one each of the subunits γ, δ and ε, which constitute the central stalk of the complex. F_1_ faces the mitochondrial matrix, and conformational changes in the F_1_ subunits catalyze the formation of ATP from ADP and Pi. The F_O_ sector (~120 kDa) is hydrophobic and embedded in the IMM. F_O_ contains a proton corridor that is protonated and deprotonated repeatedly as H^+^ ions flow down the gradient from the IMS to the matrix, causing rotation, which in turn alters the orientation and conformation of the F_1_ subunits, thus driving ATP synthesis. F_O_ consists of several copies of subunit c (8 to 10 copies in mammalian mitochondria) [178,179,180], which form a ring complex, and one copy each of the following subunits: b, the oligomycin sensitivity-conferring protein (OSCP); d and F6, which constitute the F_O_ peripheral stalk; f, the 6.8-kDa mitochondrial proteolipid (6.8PL), diabetes-associated protein in insulin-sensitive tissues (DAPIT); and g e, a and A6L, which act as F_O_ supernumerary subunits [180] (Figure 6). The c-ring is critical for the transport of protons through the F_O_ region [180]. The two F_O_ and F_1_ sectors of the F_1_F_O_–ATP synthase complex push each other in the opposite direction [181], thus transforming a proton electrochemical potential into the synthesis of ATP from ADP and P_i_. However, this complex can also act in the reverse direction, hydrolyzing ATP to pump protons and form an electrochemical potential. When the electrochemical potential of the protons is large enough to surpass the free energy of ATP hydrolysis, it drives the F_O_ sector to generate a rotary torque upon proton translocation through the c-ring to produce ATP synthesis in the F_1_ sector. Conversely, when the electrochemical potential is small, the F_1_ sector, acting as an F_1_–ATPase (hydrolyzing ATP) induces F_O_ to rotate the c-ring in the reverse direction to pump protons against the electrochemical potential [181]. Thus, the proton electrochemical potential can drive the complex to synthesize ATP and, conversely, ATP hydrolysis (as an ATP-driven motor) can lead to the transfer of protons in the opposite way. Dimers of the F_1_F_O_–ATP synthase complex have been shown to be distributed along the inner folds of the mitochondrial cristae by high-resolution transmission electron microscopy [182,183]. This dimerization and localization of the F_1_F_O_–ATP synthase at the tips of the cristae induces a strong curvature to the membrane, leading to the characteristic folded morphology of the mitochondrial cristae [183,184]. The F_1_F_O_–ATP synthase would act as a sink of protons [182], generating a H^+^ gradient higher at the cristae than in the rest of the intermembrane space. Compelling evidence has led to the novel concept that the IMM-embedded c-subunit ring of the membrane-spanning component F_O_ of the human mitochondrial ATP synthase complex, forms the mPTP [185,186,187,188], and this pore is also functional in the ATP synthase monomer, not requiring ATP synthase dimerization [189,190]. The purified reconstituted c-subunit ring of the F_1_F_O–_ATP synthase forms a voltage-sensitive channel, the persistent opening of which leads to the rapid and uncontrolled depolarization of the IMM in cells [187]. Depletion of the c-subunit hinders Ca^2+^-induced IMM depolarization as well as Ca^2+^- and ROS-induced cell death, whereas the overexpression of the c-subunit favors cell death [187]. Genetic manipulation of c-subunit expression levels by siRNA in HeLa cervical cancer cells affected the mPTP activity [185]. Knockdown of the c-subunits of the F_1_F_O_–ATP synthase reduced the mPTP opening in response to ionomycin or hydrogen peroxide and their overexpression enhanced the mPTP opening [185].

The current view of the mPTP includes a c-subunit channel embedded in the IMM (Figure 6). Mnatsakanyan and Jonas [190] have proposed a model of the F_1_F_O_–ATP synthase c-subunit channel, in which there are physiological reversible and pathological non-reversible openings of the c-subunit ring pore (Figure 6). It has been envisaged that the F_1_ sector of the F_1_F_O_–ATP synthase can act as an inhibitor of the c-subunit ring pore and it can be reversibly tilted, through a conformational change, to release the close contact between the F_1_ sector and the c-subunit pore in the F_O_ sector. This conformational change pulls away F_1_ from the mouth of the c-subunit pore to open the channel from the matrix side (Figure 6). Under certain circumstances, including during long-lasting openings of the c-subunit channel, F_1_ dissociates from F_O_, thus leading to a permanent opening, mitochondria swelling, OMM rupture, and cell death. These conformational changes can be induced by the mPTP inducers CypD and Ca^2+^ through their binding to the OSCP and β subunit of the F_1_F_O_–ATP synthase, respectively, thus inducing a conformational change in the ATP synthase peripheral stalk subunits, promoting the removal of the F_1_ sector from the top of the c-ring.

The pro-apoptotic Bcl-2 family members Bax and Bak have been suggested to function as the OMM component of the mPTP in regulating cell death [191]. The mitochondria from Bax and Bak double-deleted mouse embryonic fibroblasts (MEFs) were resistant to Ca^2+^-induced swelling, and displayed reduced OMM permeability and conductance as well as cell death [191]. In contrast, Bcl-2 (~26 kDa) and its homologue Bcl-x_L_ (~27 kDa), as well as other anti-apoptotic Bcl-2 family members, protect mitochondria by interacting with pro-apoptotic Bcl-2 members and hence prevent MOMP and subsequent apoptosis. The anti-apoptotic Bcl-2 members can also mediate the activity of the mPTP by direct interactions with regulatory components [192,193]. In addition, Bcl-x_L_ has been found to interact directly with the β-subunit of the F_1_F_O_–ATP synthase, regulating metabolic efficiency [194]. Thus, Bcl-x_L_, once thought to be present exclusively on the OMM, is now accepted to be an F_1_F_O_–ATP synthase regulator in the IMM that stabilizes the inner membrane potential [194,195].

Oligomycin has been recognized as a potent inhibitor of the mitochondrial ATP synthase since the late 1950s and 1960s [196,197], with the F_O_ sector being responsible to confer oligomycin sensitivity [197,198]. In fact, the high-resolution (1.9 Å) crystal structure of oligomycin bound to the subunit c_10_ ring of the yeast mitochondrial ATP synthase has been reported [199].

OSCP is located on top of the catalytic F1 sector (Figure 6), connecting F_1_ and the peripheral stalk, and ensuring the structural and functional coupling between F_O_ and F_1_, which is disrupted by oligomycin [200].

The Bcl-2 family of proteins have the capacity to regulate the permeability of intracellular membranes to ions and proteins. The pro-apoptotic members of the Bcl-2 family (e.g., Bax and Bid) are able to form channels in the membranes and regulate preexisting channels, whereas the anti-apoptotic Bcl-2 members have the opposing effects on membrane channel formation [201].

## 9. Edelfosine Induces Indirect and Direct Effects on Mitochondria

The inhibitor of the mPTP cyclosporin A [202] inhibited [47,82], whereas Bcl-2 or Bcl-x_L_ overexpression totally prevented [9,40,47] edelfosine-induced apoptosis in cancer cells. These results, together with the edelfosine-induced mitochondrial-mediated changes depicted in Figure 2, provide strong evidence for the major role of mitochondria in the apoptotic response triggered by edelfosine in cancer cells.

The fact that edelfosine induces Bid cleavage, generating the 15 kDa cleaved form of truncated Bid (tBid), as well as BAP31 cleavage into the p20 fragment [39,40,49], further supports the involvement of mitochondria in the pro-apoptotic action of the ether lipid. Taken together, this suggests a complex interplay between the plasma membrane, ER and mitochondria in edelfosine action. Bid is a potent pro-apoptotic Bcl-2 family member which, upon proteolytic activation by caspases 8 or 10, translocates onto mitochondria and promotes the activation of Bax/Bak, thus contributing to cytochrome *c* release [203]. BAP31 is an integral membrane protein of the ER that modulates ER-mediated apoptosis through its caspase-8-mediated cleavage into a 20 kDa fragment. This p20–BAP31 fragment prompts the discharge of Ca^2+^ from the ER and its concomitant uptake into the mitochondria, thus directing pro-apoptotic signals between the ER and mitochondria [204]. Edelfosine induces all these changes, namely, caspase-8 and -10 activation, Bax activation, cytochrome *c* release, ΔΨ_m_ loss, depletion of ER-stored Ca^2+^, and the generation of the p20–BAP31 fragment, leading to cell death [9,27,28,39,40] (Figure 2). In addition, *bax^-/-^ bak^-/-^* double-knockout SV-40-transformed MEFs were resistant to edelfosine [39], further supporting the involvement of mitochondria in the ether lipid-induced apoptosis response. These data strongly suggest the mitochondrial involvement in the pro-apoptotic effect of edelfosine, through signals generated from the death receptor-mediated extrinsic apoptotic signaling in membrane lipid rafts and from an ER stress response.

The accumulation of edelfosine in the mitochondria also raises the possibility that the ether lipid could have a direct effect on the mitochondria during the onset of apoptosis. In fact, we found that edelfosine accumulates in the mitochondria in cancer cells [81,82] and affects the mitochondria in a direct way [81,205]. Edelfosine induced swelling in isolated mitochondria from adult rat livers, indicating an increase in the mitochondrial membrane permeability [81,205]. This mitochondrial swelling was independent of ROS generation [81,205]. Furthermore, edelfosine was also found to inhibit mitochondrial respiration and decrease transmembrane electric potential on the isolated mitochondria [205]. These latter effects were also observed with the APL perifosine, together with its ability to induce mitochondrial permeability transition [205], suggesting that the above actions constitute a rather general feature of APLs. Interestingly, preincubation with the cholesterol-depleting agent methyl-β-cyclodextrin (MCD) [9,206], which disrupts membrane rafts, inhibited edelfosine-induced mitochondrial swelling in the isolated mitochondria [81], suggesting that the action of edelfosine on isolated mitochondria seems to be dependent on mitochondrial lipid rafts.

## 10. Edelfosine-Induced Apoptosis Involves F_1_F_O_–ATPase and Its Recruitment to Lipid Rafts

Oligomycin is a highly selective inhibitor of the membrane-embedded F_O_ sector (proton channel) of the F_1_F_O_–ATP synthase that binds to the rotating c-ring within the membrane and inhibits the enzyme complex [199,207,208]. We found that oligomycin prevented edelfosine-induced ΔΨ_m_ dissipation and DNA degradation in cancer cells and *Leishmania* parasites [82], suggesting a major role of the F_O_ component of the F_1_F_O_–ATP synthase in the antitumor and anti-*Leishmania* activity of the ether lipid. In fact, recent data indicate that the accumulation of edelfosine in the kinetoplast-mitochondrion, leading to ΔΨ_m_ loss and to the successive breakdown of mitochondrial and nuclear DNA, underlies the potent action of this alkylphosphocholine analog against *Leishmania* parasites [82]. Oligomycin also attenuated apoptosis and ΔΨ_m_ loss induced by erufosine in glioblastoma cells [209]. Erufosine was found to interact with the 18 kDa translocator protein (TSPO), leading to the activation of the mitochondrial apoptosis cascade [20]. Furthermore, the *Saccharomyces cerevisiae ATP7*Δ mutant, with a deletion in the gene encoding for subunit d of the stator stalk of mitochondrial F_1_F_O_–ATP synthase, which is conserved in mammalian cells [210], was resistant to edelfosine [82]. This edelfosine-resistant phenotype could be reverted by transformation with the wild-type *ATP7* gene [82]. The above evidence strongly supports the involvement of F_1_F_O_–ATPase in the killing activity of edelfosine and in the onset of APL-induced apoptosis in general.

Edelfosine affects membrane lipid organization, making membranes more fluid [211,212]. On these grounds, edelfosine could be hypothesized to make the OMM more porous and permeable, thus favoring the leakage of H^+^ ions. This would lead to the dissipation of the proton gradient, and therefore the F_1_F_O_–ATP synthase could run in reverse, that is, hydrolyzing ATP and alkalinizing the matrix by proton extrusion. Matrix alkalinization causes the mPTP opening [213], and then it could be envisaged that the F_1_F_O_–ATP synthase could promote the onset of cell death by this F_1_F_O_–ATP synthase-mediated increase in the matrix pH. This mechanism has been previously proposed for the inhibition of Bax-induced apoptosis by oligomycin in yeast and mammalian cells [214].

By proteomic analyses in lipid rafts, isolated from different hematological cancer cells through discontinuous sucrose gradient centrifugation [215,216], we found that edelfosine treatment in hematological cancer cells led to a dramatic recruitment of mitochondrial F_1_F_O_–ATP synthase to the rafts [82]. This remarkable F_1_F_O_–ATP synthase translocation into the rafts could suggest that the enzyme is either translocated to lipid rafts present at the cell surface or in the mitochondria. Several studies have reported the presence of raft-located F_1_F_O_–ATP synthase at the plasma membrane of different normal and tumor cells, having been proposed to act as a proton channel, a modulator of extracellular ATP level, or as a regulator of intracellular Ca^2+^ levels, involved in numerous biological processes, including cell migration and intracellular pH modulation [217,218,219,220,221,222,223,224].

## 11. Edelfosine Promotes Raft Translocation to Mitochondria and Presence of Raft-Like Domains in Mitochondria

The present evidence cannot discern between the translocation of F_1_F_O_–ATP synthase to lipid rafts at the cell surface or to raft domains present in the mitochondria. However, edelfosine has been shown to promote the redistribution of lipid rafts from the plasma membrane to the mitochondria, suggesting a raft-mediated plasma membrane–mitochondria link [81]. In this context, we have found a lipid raft-mediated connection between the extrinsic and intrinsic apoptotic pathways in human multiple myeloma MM144 cells [225].

The fact that edelfosine can interact with plasma membrane lipid rafts and mitochondria could led us to suggest that the ether lipid could be translocated from the plasma membrane to the mitochondria, where it would ultimately exert its pro-apoptotic activity promoting the accumulation of F_1_F_O_–ATP synthase into mitochondrial rafts, ΔΨ_m_ dissipation, cytochrome *c* release, leading eventually to cell demise. Lipid rafts were mainly located at the plasma membrane in untreated HeLa cells, as assessed by the raft marker fluorescein isothiocyanate-cholera toxin B subunit that binds ganglioside GM1 [226], mainly found in these domains [227]. Mitochondria (stained with MitoTracker Red) were observed as a widespread network in the interior of the cell. Following edelfosine treatment, the membrane rafts were gradually internalized into the cell, and colocalized with mitochondria at the time of apoptosis onset, thus unveiling a link between plasma membrane rafts and mitochondria driven by edelfosine [81]. This suggests the presence of cholesterol-rich raft-like domains in mitochondria that could be involved in edelfosine-induced apoptosis. It is interesting to note that the GD3 raft component can proceed from the cell plasma membrane to the mitochondria via a microtubule-dependent mechanism, which could be regulated by CLIPR-59, a new CLIP-170-related protein involved in microtubule dynamics [228,229,230]. Although the presence of lipid rafts in mitochondria remains a controversial issue [231,232,233,234], there is increasing evidence favoring the presence of raft-like domains in theses organelles. Lipid raft-like domains enriched in ganglioside GD3 have been found in mitochondria and are involved in apoptosis regulation [229,232,235]. It is tempting to speculate that mitochondrial membrane rafts represent specific sites where certain critical biochemical processes, including apoptosis modulation, take place. Consistently, raft disruption prevented edelfosine-induced swelling in isolated mitochondria [81] as well as mitochondrial depolarization induced by GD3 or tBid [232]. Cholesterol levels in MAMs are higher than in the rest of the ER and they influence ER–mitochondria association [106,236,237], suggesting the importance of MAMs in providing cholesterol, and likely raft components, to the mitochondria membranes. The ganglioside GM1, abundant in lipid rafts, has been reported to accumulate in the glycosphingolipid-enriched domains of MAMs, linking ER stress to Ca^2+^-dependent mitochondrial apoptosis [238]. The physical interaction between the ER and mitochondria [239] could underlie the localization of edelfosine in both the ER [40,49,76] and mitochondria [81,82] of cancer cells. This could explain how edelfosine-mediated ER stress, which releases ER-stored Ca^2+^, requires mitochondria for the apoptotic outcome [40,49,76].

## 12. Conclusions

**Figure 7:**
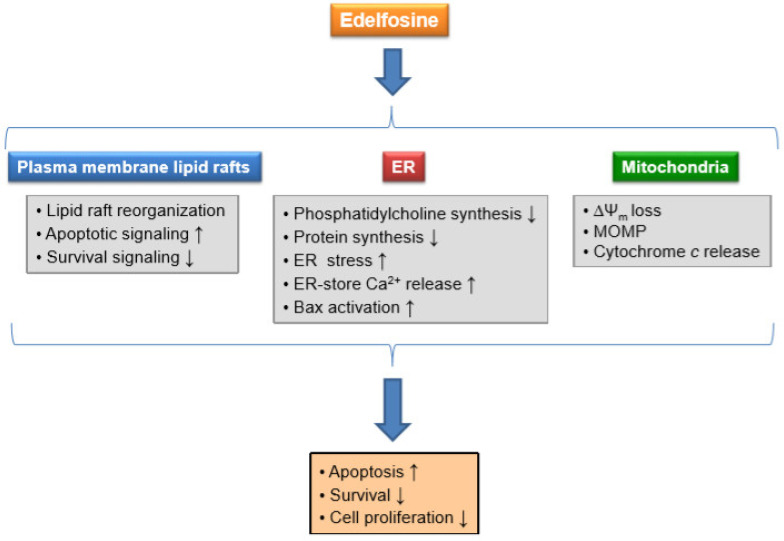
Major effects of edelfosine on lipid rafts, ER and mitochondria, and the subsequent consequences on cellular functions related to cell fate. This scheme represents several biochemical processes and cellular functions affected by edelfosine in cancer cells as discussed in the text and references therein.

This review presents a compilation and discussion of the different pieces of evidence that support the involvement and critical role of mitochondria in the antitumor action of the ether lipid edelfosine, and likely other APLs. Edelfosine accumulates in the lipid rafts, ER and mitochondria in tumor cells. The close interplay between the lipid rafts, ER and mitochondria could explain the above physical localization of edelfosine within cancer cells. Lipid rafts could be the common hypothetical link and means of ether lipid transport between the different cellular loci. Figure 7 summarizes the pleiotropic effects exerted by edelfosine on several cellular functions as a result of the drug action in a number of biochemical processes occurring in lipid rafts, ER and mitochondria. The apoptotic signaling triggered by edelfosine following raft-mediated Fas/CD95 engagement and ER stress converge through the mitochondria to render an apoptotic outcome. Mitochondria behave as the critical subcellular master regulator of cell demise. Thus, protection of mitochondria with the overexpression of Bcl-2 or Bcl-x_L_ blocks the apoptotic signals triggered by Fas/CD95 or ER stress, and prevents cell death. The affinity of edelfosine for cholesterol, a major and essential constituent of membrane rafts, could explain the above interplay between the rafts, ER and mitochondria as well as the presence of the ether lipid in the above membrane domains and organelles. The higher cholesterol level in mitochondria from tumor cells as well as in the MAMs, connecting the ER and mitochondria, together with the presence of raft domains in the mitochondria could explain the presence of edelfosine in mitochondria. Interestingly, edelfosine induces the translocation of lipid rafts from the plasma membrane to the mitochondria, pointing out a link between the cell surface and mitochondria that could also involve the ER. Although a hypothetical translocation of lipid rafts from the plasma membrane to the mitochondria, through yet unknown mechanisms, could take place, an alternative and plausible explanation could involve edelfosine-induced changes in the mitochondrial membrane (e.g., through altered cholesterol levels or distribution) resulting in the formation of raft-like structures in the mitochondria. The presence of lipid rafts or raft-like domains in mitochondria is a controversial issue, but increasing evidence supports their existence. Furthermore, the higher level of cholesterol in the mitochondria of tumor cells might suggest that cancer cell mitochondria are rather enriched in cholesterol-rich rafts that could harbor proteins and biochemical processes critical for the modulation of cell fate. In this regard, it is interesting to note that F_1_F_O_–ATP synthase is located in lipid rafts and that the c-subunit ring of the F_O_ sector constitutes the mPTP. Thus, the evidence discussed here strongly suggests that lipid rafts play a key role in the regulation of cell survival or cell death. Edelfosine, which interacts with cholesterol and accumulates in lipid rafts, promotes cell death through reorganizing the lipid rafts and their composition. In this context, edelfosine induces the recruitment of F_1_F_O_–ATP synthase into membrane rafts, and oligomycin, the potent inhibitor of the F_O_ sector, blocks edelfosine-induced apoptosis. These data support a major role for F_1_F_O_–ATP synthase in the modulation of cell death. A plausible mechanism for the pro-apoptotic effect of edelfosine in tumor cells could involve the lipid raft-mediated translocation of edelfosine from the plasma membrane to the mitochondria, where it will ultimately exert its cytotoxic activity promoting the accumulation of F_1_F_O_–ATP synthase in mitochondrial rafts, thus leading to ΔΨ_m_ dissipation, cytochrome *c* release, and eventually cell demise. The localization of edelfosine in the ER and mitochondria is in close agreement with the interaction between the ER and mitochondria, and suggests that this ether lipid could be studied as an interesting molecule to yield a further insight into these organelle interactions. This ether lipid could be used as a tool to understand the physiological and pharmacological relevance of ER–mitochondria junctions. Membrane targeting by the APL edelfosine might unveil a fascinating network of communication between the plasma membrane and organelle membranes to control cell death, as well as new insights into the role of novel membrane domains within mitochondria. These studies should help to understand membrane trafficking to mitochondria, and the link between lipid rafts and mitochondria, thus opening new avenues for novel therapeutic approaches in cancer therapy and other biomedical applications where cell death should be critically controlled. The results discussed in this review highlight the importance of cholesterol and lipid rafts in the control of cell death by mitochondria as well as in mitochondrial targeting in cancer therapy.

## Data Availability

Not applicable.

## Abbreviations

AELs	antitumor ether lipids
ANT	adenine nucleotide translocator
APAF-1	apoptosis protease-activating factor-1
APLs	alkylphospholipid analogs
BODIPY	4,4-difluoro-4-bora-3a,4a-diaza-s-indacene; boron-dipyrromethene
CypD	cyclophilin D
ΔΨ_m_	mitochondrial transmembrane potential
DiOC6(3)	3,3′-dihexyloxacarbocyanine iodide
ER	endoplasmic reticulum
ET-18-OCH_3_	1-O-octadecyl-2-O-methyl-rac-glycero-3-phosphocholine (edelfosine)
ETC	electron transport chain
IMM	inner mitochondrial membrane
IMS	inter-membrane space
MAMs	mitochondria-associated membranes
MCD	methyl-β-cyclodextrin
MEFs	mouse embryonic fibroblasts
MLN64	metastatic lymph node 64 protein
MOMP	mitochondrial outer membrane permeabilization
mPTP	mitochondrial permeability transition pore
NP-C	Niemann-Pick disease type C
NPC1	Niemann-Pick type C1 protein
OMM	outer mitochondrial membrane
OSCP	oligomycin sensitivity-conferring protein
PiC	mitochondrial phosphate carrier (PiC)
ROS	reactive oxygen species
SLC25A3	solute carrier family 25, member 3
SNAP-23	23-kDa synaptosome associated protein
STARD1	steroidogenic acute regulatory protein-related lipid transfer domain containing protein 1
STARD3	steroidogenic acute regulatory protein-related lipid transfer domain containing protein 3
tBid	truncated Bid
TSPO	translocator protein
VDAC1	voltage-dependent anion-selective channel 1
